# Dense and Nanoporous Glasses as Host Matrices to Grow Quantum Dots for Optical and Photonic Applications

**DOI:** 10.1002/smll.202410564

**Published:** 2025-02-03

**Authors:** Xue Bai, Lingzhi Wu, John J. Magan, Brian Jennings, Wei Zhou, Shenghao Wang, Yurii K. Gun'ko, Gaozhong Wang

**Affiliations:** ^1^ Innovation and Integration Center of New Laser Technology Shanghai Institute of Optics and Fine Mechanics Chinese Academy of Science Shanghai 201800 China; ^2^ School of Chemistry, CRANN and AMBER Research Centres Trinity College Dublin College Green Dublin D02 Ireland

**Keywords:** dense glass, nanoporous glass, nonlinear optics, photonic devices, quantum dots

## Abstract

Quantum dots (QDs) grown within inorganic glasses (hereafter referred to as “QD glasses”) are promising candidates for an expanding list of applications such as nonlinear optical (NLO) devices. However, lots of research into NLO properties of QDs still uses polymer‐based matrices, whose low laser damage threshold hinders practical applications. This can be explained by the difficulties typically encountered by researchers wishing to grow QDs within glass matrices. Fortunately, much progress has been made, not only as regards dense glass but also in the use of nanoporous (NP)  glass which is prepared and explored as a macro‐matrix in the growth of QDs. In situ growth techniques for the preparation of QD glasses are more appealing than ex situ methods, as the former can effectively avoid agglomeration of the QDs and the need for application of prior treatments such as ligand exchange. Here, a review of advances in growth techniques of QDs in both dense and NP glasses is provided, with a discussion on the effect of glasses on the emission nature of the grown QDs, the routes to tune emission, enhancing optical performance and, finally, potential applications of QD glasses. The overview of directions and future challenges of this area are also presented.

## Graphical abstract

This work reviews the advances in a range of QDs embedded in both dense and nanoporous inorganic glasses. Growth techniques, the effect of glasses on the emission nature of QDs, the routes to tune and enhance emission, optical performance along with potential applications of QD glasses are discussed and an overview of related directions and future challenges is also provided.

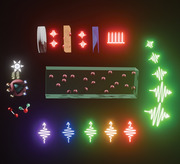

## 1 Introduction

Embedding QDs into macromatrices enhances their applicability to a wide range of applications including, but not limited to lighting, displays, photovoltaics and nonlinear optical (NLO) devices.^[^ ^1^ ^]^ Several categories of matrices have been explored to load QDs, such as ionic macrocrystals (NaCl, NaBr, KCl and so forth),^[^ ^2^ ^]^ organic macromatrices (sucrose and anthracene),^[^ ^1^, ^3^ ^]^ polymeric macromatrices (PMMA‐ poly(methylmethacrylate), thiol–ene polymers, thiol–yne polymers and some copolymers),^[^ ^4^ ^]^ nanoporous and mesoporous silica spheres,^[^ ^5^ ^]^ inorganic glasses,^[^ ^6^ ^]^ and other categories.^[^ ^7^ ^]^ Among all these macromatrices, inorganic glasses feature high thermal and chemical stability and high optical transmittance across a wide spectral range, and importantly, they display a high threshold for laser damage. The above intrinsic properties render glasses far more suitable to embed QDs for a variety of applications.^[^ ^8^ ^]^ Many techniques have been developed to grow QDs in glasses, for example the well‐known melt quenching and subsequent thermal treatment method, the ion‐exchange technique, the ion‐implantation route and so forth. Cd‐, Zn‐, and Pb‐based chalcogenide QDs, perovskite QDs, ternary alloyed QDs, and even core/shell QDs have been successfully grown in inorganic glasses.^[^ ^6^, ^9^ ^]^ Meanwhile, much effort has been devoted to investigating the growth mechanism and optical properties of obtained QD glasses. As this research area rapidly evolves, research involving dense glasses has, more recently, been complemented by incorporation of innovative work exploring NP glasses in growing QDs.

**Figure 1:**
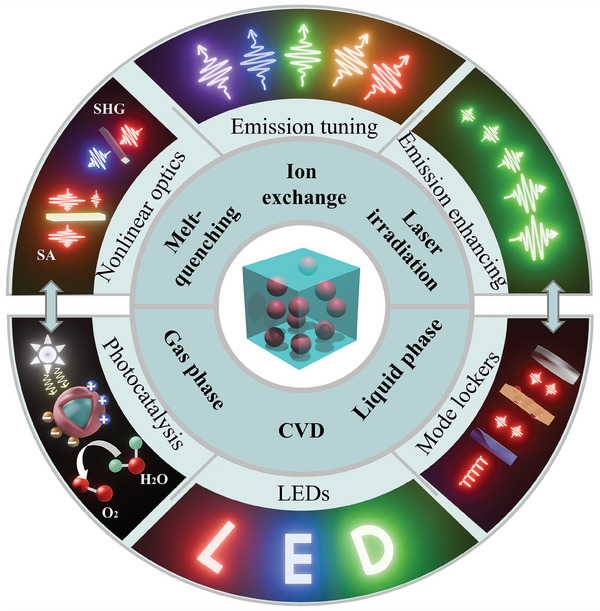
Schematic illustration of the fabrication approaches of QD glasses and their applications considered in this review.

The incorporation of QDs in both dense and NP glasses is of great importance to developing QD‐based photonic devices, particularly devices that demand high transparency over a wide spectral range, as well as high chemical and physical stability. This requires an in‐depth understanding of technological progress in this area, including the growth mechanism, the effect of glasses on the emission nature of QDs, and strategies to tune optical properties of QD glasses. In this review, we make a detailed summary of advances in growing techniques of QDs in dense and NP glasses (see **Figure** **1**), with focus on the recent research into their NLO properties, including enhancement and tunability of emission, and related photonic applications such as photocatalysis, LEDs (light‐emitting diodes) and mode‐lockers.

## 2 Techniques to Grow QDs in Glasses

Much attention up to now has focused on the growth of QDs in dense glasses and improving the optical performance of obtained QD glasses. The emergence of NP glass as host matrix has brought with it the development of several novel techniques for growing QDs. An overview of the growth techniques and the characteristic traits of QD glasses is given in **Table** **1**. The details of these techniques are elaborated in the following sections of this review.

**Table 1:** Characteristic traits associated with different growth techniques in dense and NP glasses.

Glass matrix	QD growth technique	Characteristic Traits	Refs.
	Melt‐quenching and subsequent thermal treatment	Widely investigated, applicable to grow a variety of QDs	[6, 9, 10]
**Dense**	Laser irradiation	Grow QDs in a programmable way, widely applicable	[10, 11]
**glass**	Ion implantation	Grow QDs near the top surface of the glass within a controlled depth, Controllable concentration and distribution of the QD ions, can cause damage to the glass matrix	[12]
	Ion exchange	An alternative method to control the distribution of QDs in glass	[9, 13]
**NP**	Two‐step liquid phase method	Can be applied to grow perovskite QDs at Room Temperature (RT)	[14]
**glass**	Gas phase method	Can be applied to grow Cd‐, Pb‐based QDs and even ternary Cu‐based QDs at RT or high temperature (400–450 °C)	[6, 15]
	CVD and quasi CVD technique	Can be applied to grow different QDs in NP glass, requires high temperature to vaporize chemicals.	[16]
	One‐step liquid phase method	Can be applied to grow perovskite QDs at RT	[17]

### 2.1 Fabrication of QDs in Dense Glasses

#### 2.1.1 Melt‐Quenching and Subsequent Thermal Treatment Method

**Figure 2:**
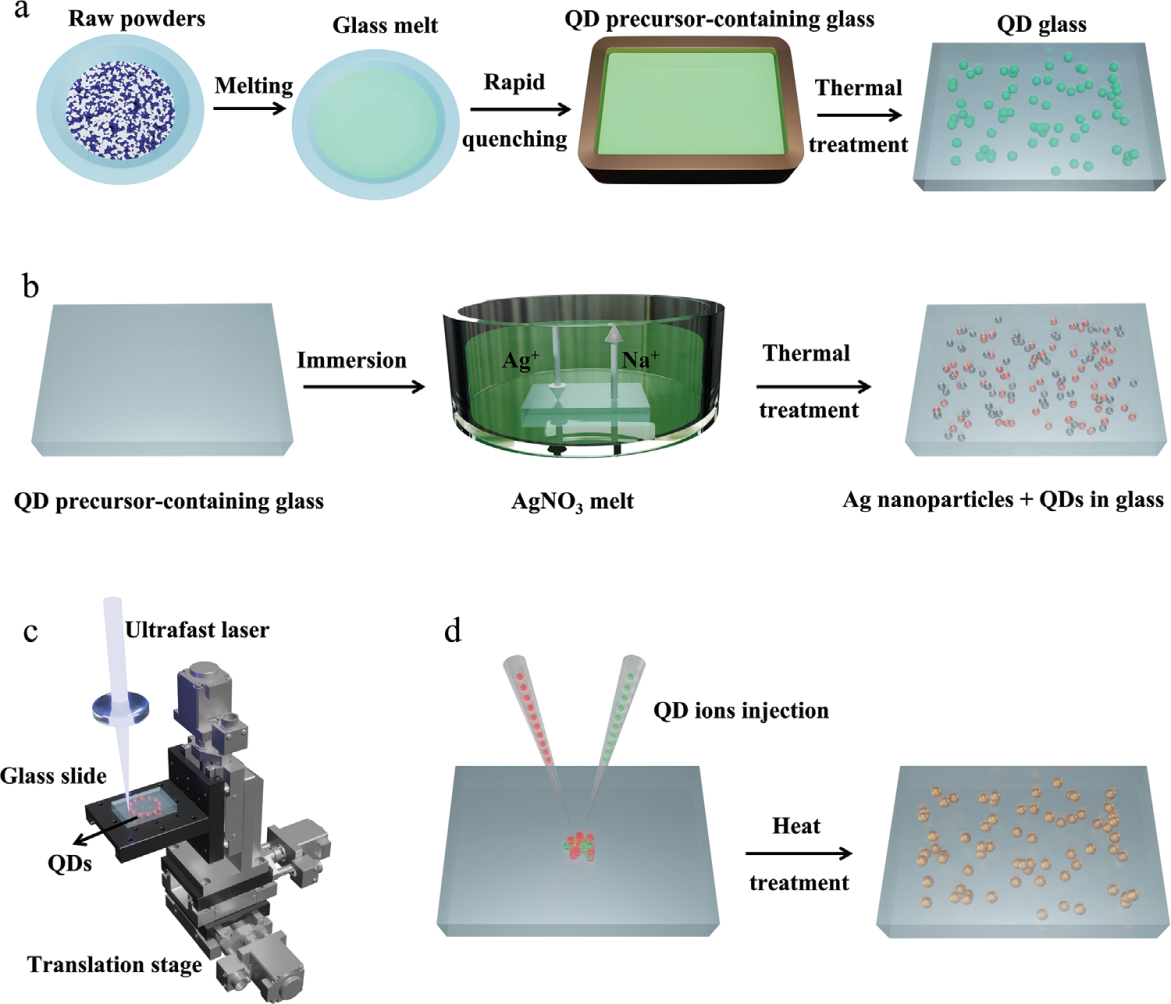
Schematic illustrations of a) melt‐quenching and subsequent thermal treatment method, b) growth of QDs in dense glasses via ion exchange, c) ultrafast laser irradiation to grow perovskite QDs in dense glass, d) ion implantation technique for growing QDs in dense glasses.

The melt‐quenching and subsequent thermal treatment method has been the most commonly used technique to grow QDs in dense glasses.^[^ ^6^, ^10^, ^18^ ^]^ As shown in **Figure** **2**a, this fabrication route involves mixing of the glass component powders and the QD‐component powders thoroughly *via* ball milling, followed by melting of the mixture at high temperature (>1000 °C) in an alumina crucible. The melts are transferred to a brass mould and rapidly cooled to RT to get a dense glass matrix. In this case, the QD‐component (cations and anions) are distributed uniformly in the glass, forming the so‐called “over‐saturated dissolution” of ions in the glass matrix. To relieve the stress in the glass, the obtained glass is normally heat treated at relatively low temperature (≈300–350 °C) for several hours. After that, thermal treatment at a more elevated temperature (450–650 °C) is conducted to allow diffusion of QD components and nucleation and growth of the QDs to take place. A prototypical example of this approach is the growth of lead sulfide (PbS) QDs in dense glass, which involves introducing the QD Pb‐component and S‐component sources into the glass powder in the form of PbO and ZnS. After melting of the mixture powder and rapid quenching to RT, Pb^2+^ and S^2−^ ions exist evenly in the as‐prepared glass which, upon high temperature heat treatment, migrate resulting in bonding of the ions and growth of the PbS QDs in the glass.^[^ ^10d^ ^]^ For the growing of CdSe QDs in dense glass, it is believed that the Se─Se contact sites are the nucleation sites in the glass, with heat treatment at high temperature (>400 °C) promoting thermal diffusion of Cd ions to the Se‐rich sites and nucleation into CdSe QDs.^[^ ^9^, ^19^ ^]^ This method has been employed to grow a variety of QDs within dense glasses, including but not limited to CdS QDs,^[^ ^6^, ^20^ ^]^ CdSe QDs,^[^ ^21^ ^]^ CdTe QDs,^[^ ^22^ ^]^ PbSe QDs,^[^ ^6^, ^23^ ^]^ PbTe QDs,^[^ ^10f^ ^]^ PbS QDs,^[^ ^6^, ^10^ ^]^ perovskite QDs^[^ ^18b^ ^]^ and even ternary Cd‐Zn‐S QDs and multi‐component Cu‐based QDs like CuInSSe QDs.^[^ ^9^, ^24^ ^]^

#### 2.1.2 Ion Exchange Method

The ion exchange method is a variation on the traditional melt‐quenching and subsequent thermal treatment technique in which material modification is achieved by substitution of monovalent Ag^+^ ions (or Cu^+^ ions) for Na^+^ ions or K^+^ ions present in the glass matrix. This process, illustrated in Figure 2b, involves dipping QD precursor‐containing glass in silver nitrate melts (copper sulphate melts) for a certain duration, or alternatively, painting one surface of the dense glass with commercial Ag paste then heating to a defined temperature (i.e., 320 °C) to promote the diffusion of Ag^+^ into the glass matrix.^[^ ^9^, ^13^ ^]^ Upon subsequent heat treatment at high temperatures, the Ag^+^ ions capture the electrons from surrounding impurities or existing nonbridging oxygens and turn first to Ag^0^ before aggregating into Ag nanoparticles. The formed Ag nanoparticles function as sites for heterogenous nucleation of QDs within the top surface of the macro inorganic glass during the heat treatment process.^[^ ^9b^ ^]^ Control of the duration of ion exchange can be used to tune the concentration of the exchanged Ag^+^ ions. The concentration of doped Ag^+^ ions dominates the manipulation of photoluminescent (PL) properties via controlling the spacing between the formed Ag nanoparticles and the QDs.^[^ ^13c^ ^]^

#### 2.1.3 Femtosecond Laser Irradiation

Femtosecond laser irradiation can be used to grow QDs in a programmed manner at specific positions in dense glass with or without the presence of noble metal nanoparticles (Ag or Au nanoparticles). Ultrashort laser pulses can provide localized thermal energy which allows for the diffusion and crystallization of QDs in dense glass (Figure 2c). When the frequency of laser irradiation is consistent with the plasmon frequency of noble metal nanoparticles present within the glass, surface plasmon resonance will produce heat around the nanoparticles, which allows for diffusion of the QD‐component ions.^[^ ^11b,e^ ^]^ Direct growth of QDs in glass by femtosecond laser irradiation relies on nonlinear multiphoton absorption‐induced heat resulting from high‐power‐density laser irradiation. Using this laser irradiation method, Cd‐, Pb‐based chalcogenide QDs, and perovskite QDs have been prepared in dense glasses.^[^ ^11^, ^25^ ^]^

Ke Sun *et al.* prepared perovskite QDs (CsPbX_3_, X = Cl, Br, or I) with tunable composition and bandgap in macro‐ inorganic 40B_2_O_3_‐15P_2_O_5_‐10Al_2_O_3_‐10ZnO‐5Na_2_O‐5K_2_O‐7Cs_2_O‐3PbX_2_‐5NaX (where X is Cl, Br, or I) dense glass via direct 3D ultrafast laser irradiation.^[^ ^11f^ ^]^ In this work, the laser beam was focused 100 µm below the surface of the glass and the focal size was ≈2 µm. Laser parameters (irradiation duration, laser power and repetition rate) were manipulated for composition tuning of the perovskite QDs. The complexation between Pb^2+^ and the halide ions is influenced by the radius and weight of the ions and hence the migration rate of halide ions in the glass matrix.^[^ ^26^ ^]^ For example, Br^−^ ion has greater complexation with Pb^2+^owing to its lighter ionic weight and smaller radius, which translates into a faster diffusion rate of Br^−^ than I^−^, and the formation of Br‐rich perovskite QDs. By extending the duration of laser irradiation, more I^−^ ions migrate into the perovskite region in the glass. Using this strategy, widely and continuously tunable color has been achieved in one single piece of Cl^−^, Br^−^ and I^−^ co‐doped glass. Due to the low formation energy of perovskite QDs, reversible 3D direct laser printing of perovskite QDs has been achieved in dense glass.^[^ ^11c^ ^]^ The emission of the grown perovskite QDs can be erased upon further femtosecond irradiation due to the partial decomposition of the grown CsPbBr_3_ QDs into PbBr_2_ and CsBr. In addition, the emission can be restored after an annealing process to reform the CsPbBr_3_ QDs. This approach is promising for information security applications.

#### 2.1.4 Ion Implantation

The growth of QDs within the top surface (up to a depth of several hundred nanometers) of glass can be accomplished via the ion implantation method.^[^ ^12b^ ^]^ The QD component ions can be injected within the top surface of dense glass (normally several hundred nanometers) with the aid of an electric field (Figure 2d). The energy and fluence of the ions can be precisely tuned to control the implantation depth and doping concentrations of the injected ions. The growth of the QDs starts upon annealing at high temperature after ion implantation. Using this technique, Pb‐based chalcogenide QDs including PbS, PbTe and PbSe QDs, as well as ZnO QDs have been grown in dense glasses.^[^ ^12a,c,d^ ^]^ It is worth noting, however, that the ion implantation process can cause serious damage to the glasses.^[^ ^9j^ ^]^

### 2.2 Fabrication of QDs in NP Glass

NP glass can be prepared using the sol‐gel method at mild temperatures using TEOS (tetraethyl orthosilicate) or TMOS (Tetramethoxysilane) as Si source or using the method of melt‐quenching and subsequent leaching in acid. It has been employed as a novel matrix to grow QDs. Ex situ methods can be used to load QDs into NP glass. For example, QDs have been incorporated into NP glass by mixing colloidal QD suspensions in a certain solvent (like toluene) with the TEOS sol (in ethanol and water), obtaining QD‐impregnated glass.^[^ ^27^ ^]^ Perovskite QDs were loaded into NP glass by soaking the glass in a corresponding QD solution.^[^ ^28^ ^]^ However, the ex situ methods cannot avoid a certain degree of aggregation of QDs, which adversely affects their optical properties. To tackle this issue, several new techniques have been explored for the direct growth of QDs in NP glasses. The pore size of the glass can be tuned from 1 to 10 nm, is comparable to the size of the QDs, thereby offering a nanoconfined growth space for QDs and a novel avenue to tailor their optical properties based on the quantum confinement effect.

#### 2.2.1 Two‐Step Liquid Phase Method

**Figure 3:**
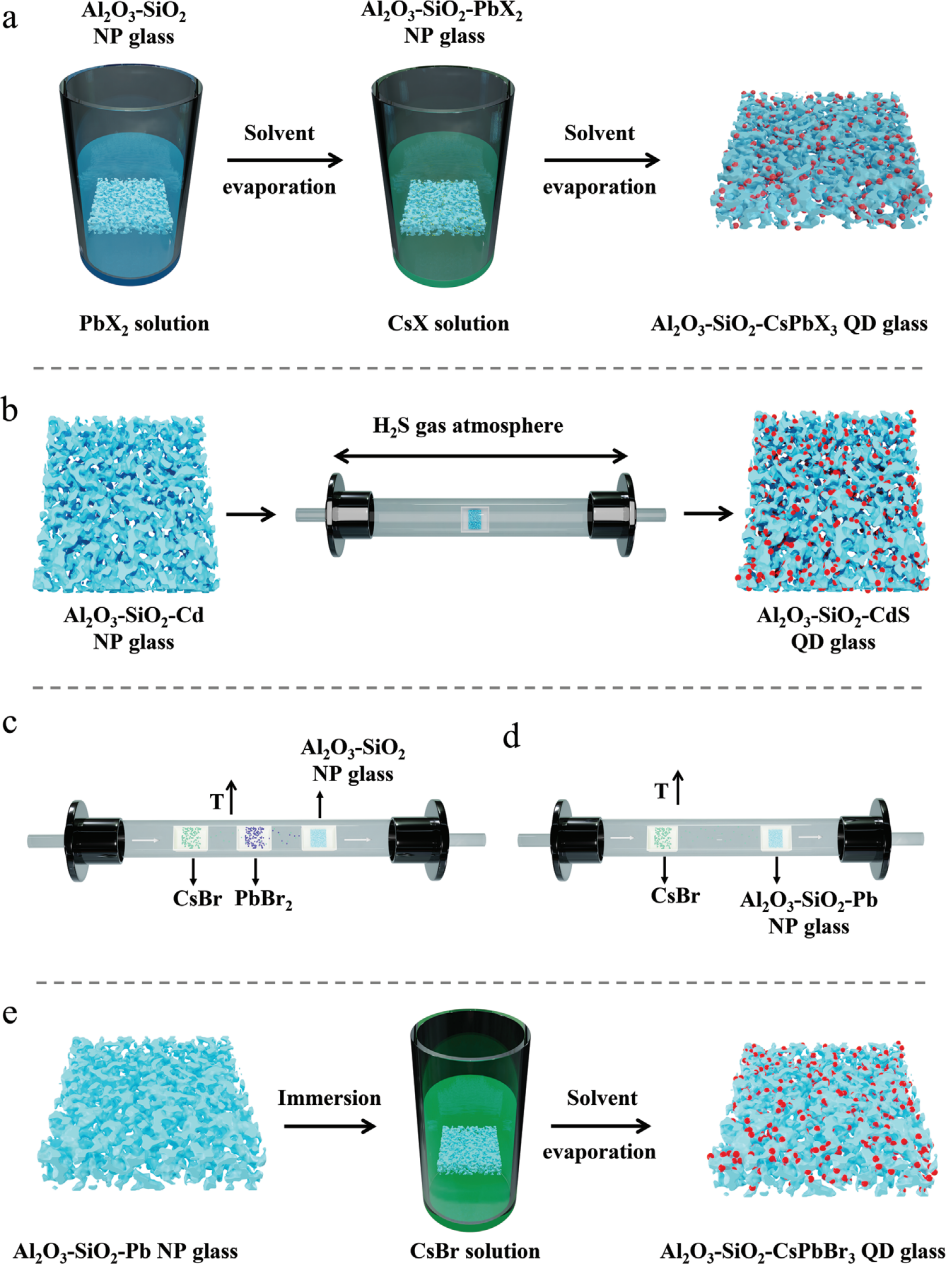
Schematic illustrations of growth of QDs in NP glass via a) two‐step liquid phase method, b) gas phase growth method, c) CVD technique using two chemical sources, d) CVD technique using a single chemical source, and e) one‐step liquid phase method.

The two‐step liquid phase method has been used to prepare perovskite QDs (CsPbX_3_ QDs, X═) in NP Al_2_O_3_‐SiO_2_ glass.^[^ ^14^ ^]^ As shown in **Figure** **3**a, the first step involves immersing the as‐prepared NP Al_2_O_3_‐SiO_2_ glass in PbBr_2_ solution in N,N‐dimethylformamide (DMF), then extracting the solvent via evaporation under vacuum at 70 °C, resulting in PbBr_2_ being left on the surface and in the nanopores of the glass. The second step consists of immersing the NP Al_2_O_3_‐SiO_2_ glass loaded with PbBr_2_ in a solution of CsBr in methanol, followed by evaporation of the solvent and nucleation and growth of CsPbX_3_ QDs in the NP glass. It is worth noting that the nucleation and growth of perovskite QDs on the surface of NP glass cannot be avoided when using this technique.

#### 2.2.2 Gas Phase Method

For the growth of Cd‐chalcogenide QDs, H_2_S can be a simpler and more affordable candidate to replace traditionally used trimethylsilyl‐based chalcogenide precursors. Meanwhile, it should be pointed out that H_2_S is a poisonous gas and requires extreme care when used. Generally, as shown in Figure 3b, cadmium acetate (as the Cd source) is introduced into the sol solution for the NP glass. After ageing at RT, and primary solvent evaporation in the oven, the formed xerogel is sintered in a furnace at high temperature (600–700 °C), resulting in NP glass containing Cd (in the form of CdO). The growth of CdS QDs happens upon exposure of the NP glass to H_2_S gas at certain temperatures.^[^ ^15^, ^29^ ^]^

The growth of QDs in NP glass via the gas phase method can also occur in the xerogel before sintering of the glass matrix. In this case, the Cd source, i.e., Cadmium acetate is added into a wet sol solution of the glass components, which is followed by evaporation of the solvent at 60 °C for two weeks. The cadmium acetate can be converted into CdO *via* heat treatment in an O_2_ atmosphere at 420 °C for 24 h. The obtained CdO‐containing gel is exposed to H_2_S gas at 120 °C or RT for 12 h, resulting in CdS QD‐containing gel. After that, the CdS QD‐containing gel is densified by sintering at 590 °C in vacuum for 12 h to get CdS QD‐containing glass.^[^ ^15a^ ^]^ In addition to the growth of CdS QDs in NP glass, this technique can also be applied to grow PbS QDs in NP glass.^[^ ^30^ ^]^

Multicomponent Cu‐based QDs have also been successfully grown in NP glass using this method. In 2000, Cu_x_S and CuInS_2_ QDs measuring 5 ± 3 nm in size were grown in NP silica glass.^[^ ^31^ ^]^ The NP silica xerogel was prepared via a sol‐gel process. The incorporation of Cu source or the Cu+In source was done by immersing the obtained porous xerogel in a solution of Cu(NO_3_)_2_ or the mixture solution of Cu(NO_3_)_2_ and In(NO_3_)_3_ for 8–12 h. After evaporation of the solvent, the Cu and In‐doped porous silica xerogel was exposed to H_2_S gas at 400 °C for 1 h. The transformation of Cu_x_S and CuInS_2_ QD‐doped xerogel to QD glass was conducted by sintering the xerogel up to 1200 °C for a certain of time. The observed absorption peaks in the 600–700 nm range are associated with the first excitonic resonance in the quantum‐confined particles.^[^ ^31^ ^]^ Following this work, a modified gas phase procedure was developed to grow Cu‐based QDs. Instead of doping the QD precursor after the formation of xerogel, the Cu or In or Ga solution (in the form of Cu(NO_3_)_2_, In(NO_3_)_3,_ and Ga(NO_3_)_3_) was introduced to the sol solution at the very beginning, with the QD precursor‐doped NP glass obtained directly after ageing of the sol solution and heating of the gel and sintering at high temperature. The Cu‐ or In‐containing NP glass was then exposed to H_2_S for a certain of time for the growth of CuInS_2_ QDs^[^ ^15e^ ^]^ and CuGaS_2_ QDs.^[^ ^15g^ ^]^

#### 2.2.3 Chemical Vapor Deposition (CVD) Technique

Using the CVD method (Figure 3c), perovskite QDs have been grown in NP Al_2_O_3_‐SiO_2_ glass. The perovskite QD component chemicals CsBr and PbBr_2_ are placed in a tubular furnace and first undergo a degassing process for 20 min. After that, the temperature is increased to 550 °C for the vaporization of the QD chemicals, followed by growth of CsPbBr_3_ QDs in the pores of the glass.^[^ ^16a^ ^]^ The low formation energy of CsPbBr_3_ QDs (≈−6.45 eV)^[^ ^32^ ^]^ results in fast nucleation and growth, filling up the nanopores in the glass. However, use of this method also results in crystallization of perovskite QDs on the surface of the glass. Without the confinement to growth of the QDs afforded by the nanopores, the larger size of surface‐grown QDs leads to emission at longer wavelengths. Even though it has not been explored, this route should also be employed to grow II‐VI QDs like CdS or ZnS QDs in NP glass.

Thus, as shown in Figure 3d, a variation on this technique involves vaporization of CsBr and permeation into the nanopores of Al_2_O_3_‐SiO_2_‐Pb glass already doped with Pb precursor. This method offers the potential to avoid QD growth on the surface of the glass.

A quasi CVD method was developed to grow Cu_2_Se QDs in NP glass.^[^ ^16c^ ^]^ In this method, NP silica glass was prepared *via* a sol‐gel process. First, porous silica xerogel was formed, followed by immersion in alcohol solution of Cu(NO_3_)_2_ and then evaporation of the solvent in air. The obtained Cu‐doped xerogel was heated to 600 °C for 1 h and further kept in flowing H_2_ gas at ambient pressure at 600 °C for 1 h. The process resulted in the formation of metallic Cu particles in the glass (CuO+H_2_→Cu+H_2_O). Then the obtained metallic Cu‐containing porous xerogel along with a calculated amount of elemental Se were placed in quartz ampoules (the amount of Se was enough to provide a partial pressure of Se vapor of ≈1 atm at 1200 °C). The growth of Cu_2_Se QDs started upon heating of the quartz ampoules to 1200 °C. It was believed that the formed Cu_2_Se QD glass was a quite promising material for saturable absorbers.^[^ ^16b,c^ ^]^

#### 2.2.4 One‐Step Liquid Phase Method

As already mentioned, the two‐step liquid phase method typically results not only in the formation of QDs in the nanopores of the glass, but also nucleation of QDs on the glass surface. One method to overcome this issue is via a one‐step liquid phase method. The QD‐component Pb can be incorporated into the sol solution of Al(lact)_3_ and TEOS, producing NP Al_2_O_3_‐SiO_2_ glass containing Pb. The Pb‐containing NP Al_2_O_3_‐SiO_2_ glass can then be immersed in CsBr solution in methanol for several minutes for the growth of CsPbBr_3_ QDs in the nanopores of the glass (Figure 3e).

Recently, a one‐step solution method has been developed for the growth of PbS QDs in mesoporous aluminosilicate glass using Na_2_S solution as the sulfur source by Junwei Chen and coworkers.^[^ ^17b^ ^]^ The PbO‐Al_2_O_3_‐SiO_2_ was first prepared via a sol‐gel procedure, which was conducted by introducing lead acetate trihydrate into the sol solution of L‐aluminum lactate and TEOS. Sintering at 600 °C of the dried gel (obtained after evaporation of solvent in the oven at 100 °C) in the muffle furnace resulted in PbO‐Al_2_O_3_‐SiO_2_ mesoporous glass. The preparation of PbS‐ Al_2_O_3_‐SiO_2_ QD glass was done by immersing the as‐prepared mesoporous PbO‐Al_2_O_3_‐SiO_2_ glass in Na_2_S solution (tris (hydrochloric acid) buffer used as the solvent) for 30 min. As growth of PbS QDs proceeded, the glass was observed to change from colorless to brown then to black. In addition, it has been claimed that the nanosized pores present in the mesoporous glass limit the overgrowth of PbS QDs, so that PbS QDs grow in a nanoconfined manner. This method could also be applicable to growing CdS QDs in NP glass.

Another one‐step liquid phase method was proposed by Kenji Shinozaki and Naoki Kawano to synthesize organic‐inorganic perovskite QDs in NP glass.^[^ ^17^ ^]^ The NP glass was obtained using a melt‐quenching and subsequent leaching in 1N‐HNO_3_ for 24 h. The obtained NP glass and the as‐prepared perovskite powder (C_6_H_5_C_2_H_4_NH_3_)_2_PbBr_4_^[^ ^33^ ^]^ were mixed in DMF under vacuum to impregnate perovskite into the nanopores of the glass. Evaporation of the DMF resulted in organic‐inorganic perovskite QDs in glass.

## 3 Optical Properties of QD Glasses

### 3.1 Influence of Glass Matrices on Emission Nature of QDs

QD glasses, especially the II‐VI chalcogenide QDs (CdSe, CdS, ZnS) and IV‐VI chalcogenide QDs (PbSe, PbS), typically exhibit lower photoluminescent quantum yield (PLQY) than their colloidal counterparts. QDs tend to exhibit a high defect density due to their large surface‐to‐volume ratio. For colloidal QDs, the binding of long‐chain ligands on the surface and the coating of a shell with small lattice mismatch to the core QD can favor the passivation of surface defects and enhance PLQY. In the case of QDs embedded in glass and the aforementioned preparation techniques, it is difficult to include long‐chain ligands into the growth process and realize effective binding to the surface of QDs or any post‐synthesis treatment to improve QD optical properties. It is becoming increasingly apparent that the glass matrix itself plays an important role  in optical properties of the QDs embedded within it.

**Figure 4:**
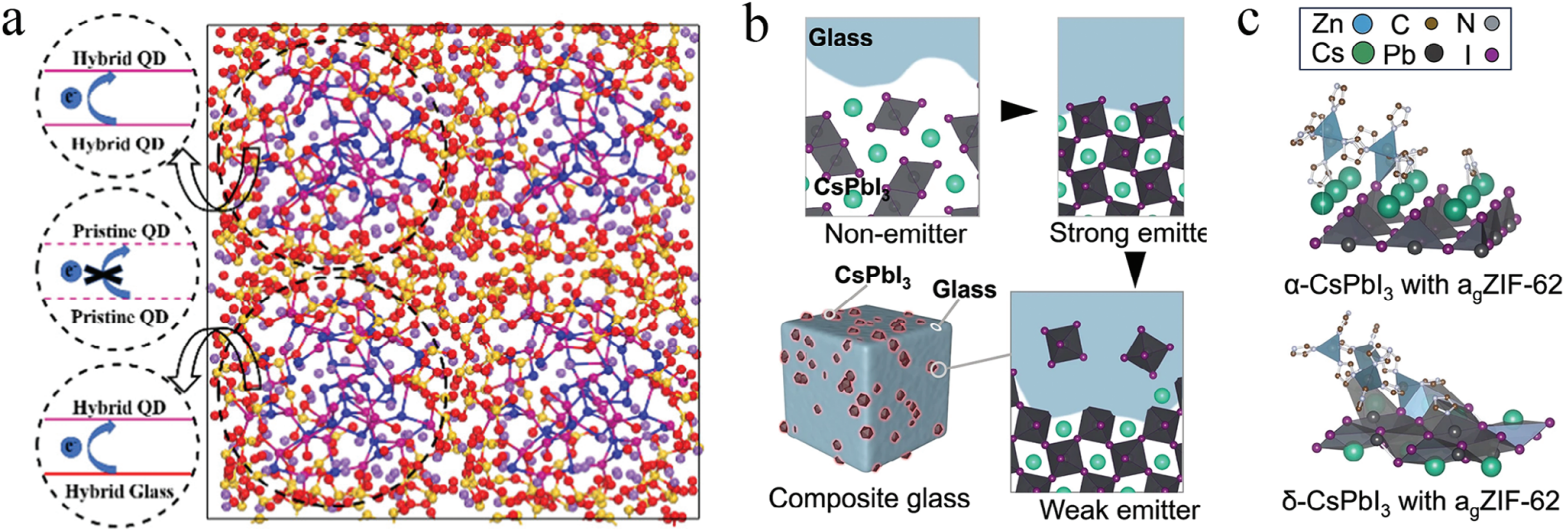
a) The configuration from the *ab* initio molecular dynamics (AIMD) simulations of CdSe QD glasses. Reproduced with permission.^[^ ^35^ ^]^ Copyright 2021, American Chemical Society. b) Schematic diagram of the CsPbI_3_ phase transition and evolution of the interfacial atomic structures during sintering and c) schematic diagram of the DFT calculation for composites with different CsPbI_3_ crystal phases. Reproduced with permission.^[^ ^37^ ^]^ Copyright 2023, Springer Nature.

The first proposed mechanism is that a high density of surface defects is formed at the interface of the grown QDs and the glass matrix. For Cd‐based QDs in glass, one of the most significant optical features is a broadened emission peak relative to their colloidal counterparts.^[^ ^34^ ^]^ It is believed that the glass matrices have a great influence on defect formation in QDs, with ramifications for their optical properties including lower PLQY. It has been suggested that emission is from the hybrid system of QDs and glass, rather than the intrinsic emission from the QDs themselves, since the existence of pristine QDs in such a system is not realistic.^[^ ^35^ ^]^ Using density functional theory (DFT) calculations, Li and coworkers characterized the electronic structures of CdSe QDs embedded in xNa_2_O‐(1‐x)SiO_2_ glasses (x = 0, 0.25, 0.33, and 0.5) (**Figure** **4**a).^[^ ^35^ ^]^ Their results demonstrated that introduction of Na_2_O into the SiO_2_ system causes the breaking of Si─O, O─Na, and Cd─Se bonds and the formation of Cd─O and Se─Na bonds. The pure QDs are less likely to exist in the Na_2_O‐SiO_2_ glass. This provides us a new perspective to elucidate the emission nature of QD glasses. Further work, based on time‐dependent density functional theory (TDDFT) results of CdSe QDs embedded in glasses, described the presence of non‐bridging oxygen atoms and undercoordinated Se atoms at the interface of the QD and the inorganic glasses. These states induce the localization of the hole wavefunction and the formation of low‐energy excited states with weak oscillator strength (optically weak). These states provide greater opportunities for non‐radiative photophysical processes, resulting in a relatively low PLQY.^[^ ^36^ ^]^ Their model was also supported by experimental results.

Recently, it has also been found experimentally that there is interfacial alloying between perovskite QDs and the glass (Figure 4b,c).^[^ ^37^ ^]^ It is also found that the inorganic glass has a significant influence on the optical properties of in situ grown QDs.^[^ ^38^ ^]^ Although much progress has been made, the precise nature of interfacial alloying between QDs and glassis not yet fully understood. More fundamental work remains to be done to elucidate the emission mechanism of *in situ* obtained QD glasses.

### 3.2 Emission Tuning of QD Glasses

#### 3.2.1 Emission Tuning of Dense QD Glasses

Several methods have been developed for the growth of QDs in dense glasses, each with different associated strategies for tuning the emission range. These are summarized in **Table** **2**.

**Table 2:** Parameters used for emission tuning in dense glasses.

Growing method	Emission tuning techniques	Effects	Refs.
**Melt quenching**	Thermal treatment temperature and duration	Growing QDs in larger size	[10, 39]
**and**	Doping ions in QDs	Introducing intra‐band states	[9, 34, 39, 40]
**subsequent**	Simultaneous doping	Energy transfer	[41]
	rare earth ions in glass	Influence on QD growth	[40, 42]
**thermal treatment**	Doping glass modifier	Changing T_g_ (glass transition temperature) of Glass matrix	[43, 44, 45]
	Tuning glass composition	Changing T_g_ of Glass matrix	[46]
**method**	Growing alloyed QDs	Changing bandgap of QDs	[9, 24, 47]
**Ion implantation**	Annealing temperature and duration	Increased QD size	[12a,c]
**Laser irradiation**	Laser irradiation time and power density	Increased QD size	[11, 48]
**Ion exchange**	Thermal treatment temperature and time	Increased QD size	[9b]
**method**	Ag^+^ concentration	Nucleation sites	[13b]

**Figure 5:**
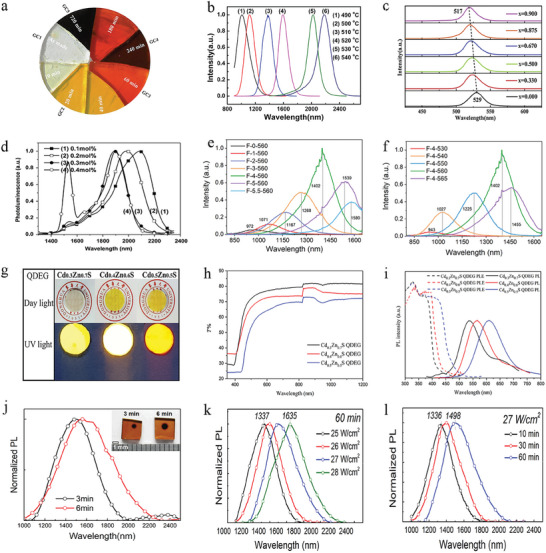
a) Photograph of CdSSe QD glasses after different heat treatment durations. Reproduced with permission.^[^ ^39b^ ^]^ Copyright 2018, Elsevier. b) Redshifted PL spectra of PbS QD glasses thermally treated at various temperatures. Reproduced with permission.^[^ ^39d^ ^]^ Copyright 2015, The American Ceramic Society and Wiley Periodicals, Inc, John Wiley and Sons. c) PL spectra of CsPb_1‐x_Zn_x_Br_3_ QD glasses with different molar ratios between Pb and Zn. Reproduced with permission.^[^ ^40h^ ^]^ Copyright 2019, Elsevier. d) Normalized PL spectra of PbS QD glasses with different Er_2_O_3_ concentrations heat‐treated at 500 °C for 20 h. Reproduced with permission.^[^ ^40b^ ^]^ Copyright 2015, The American Ceramic Society and Wiley Periodicals, Inc, John Wiley and Sons. e,f) Redshifted PL spectra of PbSe QD glasses with increasing doping of NaF in the glasses and redshifted PL spectra of PbSe QD glasses with the same doping of NaF heat treated at increasing temperatures. Reproduced with permission.^[^ ^45^ ^]^ Copyright 2022, Elsevier. g–i) Photographs of the ternary Cd‐Zn‐S QDs in glass with varying Cd:Zn ratios and the corresponding transmission and PL spectra. Reproduced with permission.^[^ ^50^ ^]^ Copyright 2022, The American Ceramic Society, John Wiley and Sons. j) PL spectra from the black spots (inset) of the glasses upon 3 and 6 min laser irradiation. Reproduced with permission.^[^ ^11b^ ^]^ Copyright 2022, Elsevier. k,l) PL spectra of glass samples irradiated at varied laser power density for 60 min and at 27 W cm^−2^ for different exposure times, respectively. Reproduced with permission.^[^ ^11b^ ^]^ Copyright 2022, Elsevier.

For the most commonly used melt‐quenching and subsequent thermal treatment method, the basic principle for tuning the emission range is based on the manipulation of QD size via changing the thermal treatment temperature and duration (**Figure** **5**a,b).^[^ ^10^, ^39^ ^]^ In general, elevating the treatment temperature and extending the treatment duration facilitate QD growth, producing larger sized QDs.^[^ ^10d^ ^]^ The higher treatment temperature favors fast diffusion of QD component ions, and longer thermal treatment duration benefits the continuous diffusion of QD component ions in the glasses. The most significant feature of QDs is the quantum confinement effect, with larger QD size associated with a shift in the emission range of the QDs to longer wavelengths. This tuning strategy is also applicable to the ion implantation method, by increasing annealing temperature^[^ ^12a^ ^]^ and extended annealing duration^[^ ^12c^ ^]^ promoting the growth of QDs of larger size. The composition of the dense glass matrix also affects the growth and thus, the emission range of grown QDs. For example, PbSe QDs were grown in germanosilicate dense glasses via melt‐quenching and subsequent thermal treatment method, with nominal compositions (mol%) of the dense glasses defined as (50‐x)SiO_2_‐xGeO_2_‐25Na_2_O‐10BaO‐5Al_2_O_3_‐1.2ZnSe‐0.6PbO, x = 0‐40 mol%.^[^ ^46^ ^]^ The emission peak shifted from 1122 to 2615 nm when the content of GeO_2_ increased from 0 to 40 mol% under the same thermal treatment procedure. It has been found that the glass transition temperature T_g_ of the germanosilicate dense glasses decreased from 471 to 442 °C with increasing GeO_2_ concentration in the glass, which translated into enhanced diffusion of QD precursor ions in the glasses with higher GeO_2_ concentration. Under the same thermal treatment temperature (470 °C), enhanced diffusion of QD precursor ions resulted in more rapid growth of QDs and larger QD size (from 2.48 to 12.54 nm) over a period of 10 h. Thus, a corresponding redshift was observed in both the absorption and emission peaks.

Another commonly used route is doping other ions in the QDs by mixing the dopant ions with the glass components for melt quenching. The doped ions (Ti^2+^, Mn^2+^, Sn^2+^, Zn^2+^, Cd^2+^, and so forth) introduce intra band states (between the valence band and conduction band of the corresponding QDs) which can function as recombination sites for the charge carriers or bring about alteration to the bandgap of the QDs.^[^ ^9^, ^34^, ^39^, ^40^ ^]^ As shown in Figure 5c, Zn has been incorporated into CsPbBr_3_ QDs in dense glasses via melt quenching and subsequent thermal treatment method. The emission peak of the obtained CsPb_1‐x_Zn_x_Br_3_ QD glasses underwent a blueshift from 529 to 517 nm as the molar ratio of doped ZnBr_2_ to the total doped ZnBr_2_ and PbBr_2_ increased from x = 0 to x = 0.9.^[^ ^40h^ ^]^ The blueshift of both absorption and emission spectra can be explained by the shrinkage of the QD unit cell due to the addition of smaller Zn^2+^ compared to Pb^2+^ into the QD crystalline unit, and broadening of the bandgap of the QDs.^[^ ^40^, ^49^ ^]^

Similarly, the emission peak of Pb_1‐x_Ti_x_Se QD glasses (heat treated at 530 °C for 10 h) blueshifted from 1890 to 1625 nm gradually as the doping concentration of Ti^2+^ in the PbSe QDs increased from 0 to 0.5 mol%. It is claimed that the incorporation of Ti^2+^ has a slight influence on the size of the as‐grown QDs, which may cause a blueshift in the absorption spectra. However, the main reason given for the observed blueshift is a change in bandgap upon the introduction of Ti^2+^ into PbSe QDs.^[^ ^40e^ ^]^

Doping with rare earth ions (like Er^3+^ and Nd^3+^) together with QDs in glass systems can cause energy transfer between these species, which gives rise to a change in the optical properties of QDs grown in the glasses.^[^ ^41^ ^]^ The lifetimes of rare earth ions are generally much longer than those of QDs, which facilitates the energy transfer to QDs. The doped rare earth ions can also influence the precipitation of QDs in dense glass matrices. It has been found that the concentration of various rare earth ions including Er^3+^, La^3+^, and Ho^3+^ have similar effects on the growth of QDs. For example, when Er_2_O_3_ concentration increased from 0.1–0.4 mol%, the size of the grown PbS QDs decreased from 5.4 to 4.8 nm when heat treated at 500 °C for 20 h, with a corresponding blueshift from 2084 to 1893 nm in the emission peak (Figure 5d).^[^ ^40^, ^42^ ^]^ It was believed that PbS QDs may nucleate on clusters of Er^2+^ ions via bridging oxygens in the glass. Increasing the Er_2_O_3_ concentration induced more nucleation sites in the glass which, combined with the limited amount of Pb and S ions in the vicinity of the nucleation sites, translated into smaller PbS QDs grown in the glass and an observed blueshift in the emission spectrum.

In addition, it has been found that doping of metal fluoride (such as CaF_2_ and NaF) in the glass network helps to break the tight bridging oxygen bonding in the glass and modify the connectivity of the glass network as evidenced from the Raman spectra and the ^19^F MAS (magic‐angle spinning)‐NMR (nuclear magnetic resonance) spectra.^[^ ^43^ ^]^ The breaking of the bridging oxygen bonds enhances ion mobility during the heat treatment process, which has great influence on the precipitation of QDs like perovskite QDs and PbS QDs in glass matrices. For example, as the NaF concentration increases, a redshift in the emission peak from 972 to 1580 nm of PbSe QDs can be observed for PbSe QDs (Figure 5e).^[^ ^45^ ^]^ In addition, in this work, the emission range of the PbSe QD glass with fixed doping concentration of NaF (4 mol%) was also managed to tune from 943 to 1455 nm by using elevated thermal treatment temperature (Figure 5f). Other glass additives also have a similar effect on the crystallization of QDs in glass matrices.^[^ ^44^ ^]^

Growing alloyed QDs in a dense glass matrix is also an important way to tune emission. Until now, alloyed Cd‐Zn‐S QDs, Cd‐Zn‐Se QDs, Cd‐Se‐S QDs, Cd‐Zn‐Se‐S QDs, Pb‐Sn‐Se QDs, and perovskite QDs have all been grown in the dense glass matrices.^[^ ^9^, ^24^, ^47^ ^]^ As shown in Figure 5g–i, increasing the doping ratio between Cd and Zn results in different fluorescent intensity under UV light, a redshift in both absorption and PL  spectra and the difference of the transmission spectra.^[^ ^50^ ^]^ In this work, the emission peak shifted from 537 to 614 nm upon increasing the ratio between Cd and Zn in the glass matrix. The increasing Cd doping concentration induced enhanced photon scattering and caused an observed decrease in transmittance of the samples.^[^ ^50^ ^]^ P. D. Persans *et al.* have grown Cd‐Zn‐S QDs in dense glass via melt‐quenching and subsequent thermal treatment technique.^[^ ^9c^ ^]^ In this work, a blueshift in the absorption spectra was observed as the growth time was increased from 30 min to 2 h at 650 °C (thermal treatment temperature). The authors explained this effect by the incorporation of Zn in the CdS QDs. It has been found that upon thermal treatment, nucleation and growth of CdS QDs proceeds first, with Zn present in the glass matrix being incorporated into the grown CdS QDs and turning them into Cd‐Zn‐S QDs and inducing a widening of the bandgap.^[^ ^9c^ ^]^ In the case of emission tuning of perovskite QDs grown in dense glasses, in addition to modification of QD size, anion composition is an alternative way to tune their fluorescence.^[^ ^51^ ^]^

As shown in Figure 5j–l, for laser power irradiation method, changing the laser duration and laser power density can be effective ways to tune the emission range of QDs grown in glass matrix.^[^ ^11^, ^52^ ^]^ The PL peak undergoes a redshift upon increasing the laser power density (perovskite QD glasses)^[^ ^48^ ^]^ and prolonging laser duration (Pb chalcogenide QD glasses).^[^ ^11b,e^ ^]^ The higher laser power density translates into elevated temperature, thus promoting the diffusion of QD component ions in the glass. Similar to the melt‐quenching and subsequent thermal treatment method, prolonging the laser duration favors the continuous diffusion of QD component ions for larger sized QDs. The laser irradiation also impacts the nucleation of the perovskite QDs in glasses. Under laser irradiation, the color of the irradiated regions turns from brown to yellow due to the growth of CsPbBr_3_ QDs in these regions, and strong green emission can be observed as well. Increasing the laser power density and prolonging the irradiation duration results in a larger laser‐modified region. The emission peaks of the obtained samples varied slightly with laser power density and exposure time within a range from 512 to 516 nm, indicating a small variation of QD size grown under different laser power intensities and exposure durations. Notably, longer irradiation duration resulted in blueshifted emission peaks, which can be explained by classical nucleation theories, extended exposure time under the same laser power density gives rise to more QD nuclei. Due to the limited amount of QD precursors present in the vicinity of the formed nuclei, the QDs would grow to a smaller size.^[^ ^11^, ^53^ ^]^ Use of a laser source could be a delicate and effective avenue to tune the emission of QD glasses in terms of the nucleation and size of the grown QDs.

For ion exchange method, not only does heat treatment temperature play a part in tuning the emission range via manipulating of the size of the QDs, Ag^+^ ions present in the glass matrix function as heterogeneous nucleation sites for the QDs, which has a great impact on the size of the QDs. In one study, it was found that generally, the size of the QDs that form in an Ag^+^ present area is larger than that of the QDs that form in the Ag^+^‐free area in the glass.^[^ ^13b^ ^]^ As the concentration of Ag^+^ was increased, the size of the QDs became smaller due to the presence of more nucleation sites for QDs, giving rise instead to higher QD concentration.

#### 3.2.2 Emission Tuning of NP QD Glasse

NP glass features tunable pore size and the porous nature can be maintained after the growth of QDs as they only occupy a certain volume of the nanopores, which offers potential novel strategies to adjust emission range as compared to dense QD glasses.

**Figure 6:**
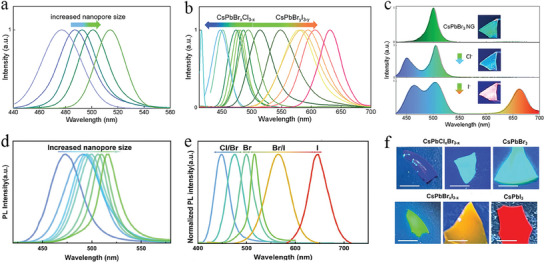
a,b) PL tunability of perovskite QDs in NP glass via changing the pore size and halide elements ratio. Reproduced with permission.^[^ ^14^ ^]^ Copyright 2022, John Wiley and Sons. c) PL tuning by partial halide exchange of perovskite QDs in NP glass. Reproduced with permission.^[^ ^14^ ^]^ Copyright 2022, John Wiley and Sons. d) Redshifted PL spectra of perovskite QD glasses upon increasing pore size. Reproduced with permission.^[^ ^16a^ ^]^ Copyright 2024, John Wiley and Sons. e) Emission tuning of perovskite QD glasses by using mixed precursor powders in a CVD technique and f) photographs of the obtained perovskite QD glasses. Reproduced with permission.^[^ ^16a^ ^]^ Copyright 2024, John Wiley and Sons.

The nanosized pores can impose confinement effects on the growth of QDs, offering an alternative route to tune the emission of QD NP glass. Hu et al. prepared perovskite QDs in NP Al_2_O_3_‐SiO_2_ glass via a two‐step liquid phase method.^[^ ^14^ ^]^ The pore size of NP Al_2_O_3_‐SiO_2_ glass can be tuned in a range from 3 to 8 nm by changing the ratio between Al and Si of NP glass (xAl_2_O_3_‐(100‐x) SiO_2_ (x = 2, 4, 6, 8, 10). The PL peak of the formed CsPbBr_3_ QDs redshifted from 478 to 514 nm correspondingly as shown in **Figure** **6**a.^[^ ^14^ ^]^ In addition to the morphology, the chemical composition of QDs also plays an important role in emission tuning. In a recent report, it was found that immersing NP glass in various mixed precursor solutions of different halide ions (CsCl, CsI, and CsBr), tuned emission from 420 to 630 nm as shown in Figure 6b. For perovskite QD NP glasses, post halide anions replacement is one of the effective avenues for tuning their emission, an option that remains unavailable for perovskite QD dense glasses. In this work, the anion exchange was done by simply soaking the obtained perovskite QD NP glass in corresponding anion solutions. The emission changed from green for CsPbBr_3_ QDs to blue and reddish in the case of CsPb(Br/I)_3_ and CsPb(Cl/Br/I)_3_ QD NP glasses (Figure 6c).^[^ ^14^ ^]^

Similar results have been reported recently. For example, the pore size has been tuned from 1.4 to 5 nm by changing the molar ratio between Al and Si in NP Al_2_O_3_‐SiO_2_ glass, resulting in a modulated emission peak from 473 to 516 nm (Figure 6d).^[^ ^16a^ ^]^ In this work, a CVD technique was employed to grow perovskite QDs in NP Al_2_O_3_‐SiO_2_ glass, achieving a wide range of emission adjustment by using more than one halide powder (the mixture of CsPbX_3_, X = Cl, Br, I) as illustrated in Figure 6e,f.^[^ ^16a^ ^]^

Other parameters including QD precursor doping concentration, growth time and so forth greatly influence emission tuning of QD NP glass. When gas phase method (H_2_S gas) was used to grow CdS QDs in NP glass, extending the growth time resulted in larger QD size and a corresponding redshift in the absorption peak.^[^ ^29^ ^]^ Increasing the doping concentration of Cd in the glass matrix gave rise to a similar enlarging of CdS QDs grown in the NP glass.^[^ ^15a,c^ ^]^

For II‐VI QDs, alloyed QDs like Cd‐Zn‐S QDs and Cd‐Zn‐Se QDs have not yet been successfully grown in nanoporous glass. But it could represent an effective route to tune the emission range of QD NP glass as emission spectrum of QDs is affected by chemical composition.

### 3.3 Emission Enhancing of QD Glasses

QDs were first discovered in inorganic silicate glass.^[^ ^54^ ^]^ In order to enhance the optical properties of QDs for practical applications, quite a few techniques have been developed to prepare colloidal QDs with long‐chain ligands on the surface of the QDs. The passivation effect of ligands to QD surface defects results in improved PLQY.^[^ ^55^ ^]^ In addition, the heterostructured type I core/shell colloidal QDs display outstanding PLQY and stability thanks to the combined effects of surface defect passivation and confinement of photon‐induced excitons in the core of the QDs.^[^ ^56^ ^]^ However, in a dense glass, the absence of long‐chain ligands attached to the QD surface results in a compromised PLQY. The central challenge posed by this limitation has spurred considerable effort to develop strategies to improve the optical performance of QD glasses.

#### 3.3.1 Localized Surface Plasmonic Effect of Noble Metal Nanoparticles

**Figure 7:**
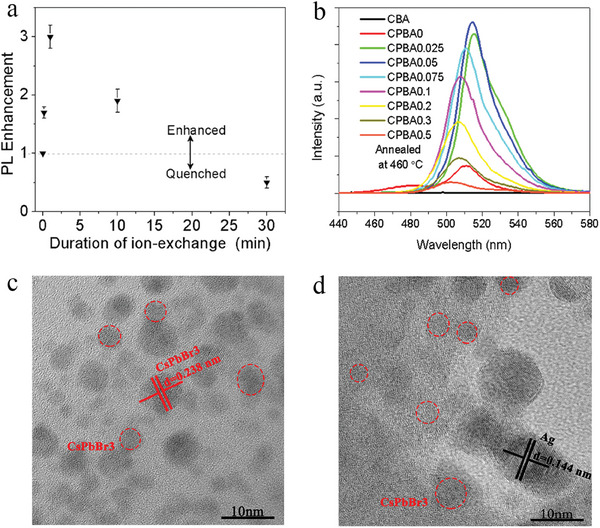
a) Emission enhancement of CdS QD glass versus duration of Ag^+^ ion‐exchange followed by heat treatment at 400 °C for 10 h. Reproduced with permission.^[^ ^13c^ ^]^ Copyright 2013, The American Ceramic Society, John Wiley and Sons. b) PL spectra of CsPbBr_3_ QD glasses with different Ag^+^ concentrations. Reproduced with permission.^[^ ^59^ ^]^ Copyright 2019, Optica Publishing Group. c,d) TEM images of undoped CsPbBr_3_ QD glass and the CsPbBr_3_ QD glass doped with 0.1 mol% Ag^+^ respectively (CsPbBr_3_ QDs were marked with red circles and red lines, the Ag nanoparticles are marked with black lines). Reproduced with permission.^[^ ^60c^ ^]^ Copyright 2019, The American Ceramic Society, John Wiley and Sons.

The first avenue that has been investigated is introducing Ag nanoparticles in QD glasses. The plasmon‐enhanced luminescence between colloidal QDs and noble metal nanoparticles has been extensively studied.^[^ ^57^ ^]^ If the QDs are located within 30 nm of the noble metal nanoparticles, their fluorescence will be dramatically enhanced, while if the distance between them is too close, the emission would be quenched severely due to energy transfer between these two nano species.^[^ ^58^ ^]^ In 2013, Kai Xu and Jong Heo fabricated Ag nanoparticles and CdS QDs glasses by means of Ag^+^ ion exchange.^[^ ^13c^ ^]^ In this work, CdS QDs were grown in dense glass using a melt‐quenching and subsequent thermal treatment method. Then Ag^+^ was incorporated into the CdS QD glass upon dipping the obtained QD glass in AgNO_3_ melt for different durations (10 s, 1,10, and 30 min) and the substitution of Ag^+^ for Na^+^ in the glass matrix. After heat treatment at high temperatures (350–450 °C) for 10 h, the Ag^+^ ions capture electrons from impurities or nonbridging oxygens present in the glass matrix and form Ag nanoparticles. The concentration of Ag^+^ ions incorporated in the QD glass can be tuned by manipulating the dipping duration of the CdS QD glass in the AgNO_3_ melt. When the dipping duration was 30 min, the fluorescence of CdS QDs in the glass was quenched. When the dipping duration was fixed at 10 s, a factor of five enhancement of the fluorescence was achieved thanks to interaction with the formed Ag nanoparticles in the glass matrix as shown in **Figure** **7**a.^[^ ^13c^ ^]^ Later on, Zhousu Xu *et. al*. precipitated Ag NPs and CsPbBr_3_ QDs simultaneously by the means of a melt‐quenching and subsequent thermal treatment procedure (using Ag_2_O as the Ag^+^ source along with other ingredients).^[^ ^59^ ^]^ It has also been found that the concentration of Ag NPs precipitated in the glass exerts a great influence on the emission enhancement of CsPbBr_3_ QDs upon the plasmonic near‐field effect (Figure 7b). A factor of five enhancement in the total fluorescence was achieved with 0.05 mol% Ag_2_O.^[^ ^59^ ^]^ Similar results have been found with perovskite QDs and Ag nanoparticles embedded glasses.^[^ ^60^ ^]^ Transmission electron microscopy (TEM) images clearly showed the presence of Ag nanoparticles in the vicinity of CsPbBr_3_ QDs formed within a glass matrix (Figure 7c,d).^[^ ^60c^ ^]^ It could be expected that by careful tuning of the concentration, a localized surface plasmonic effect of the noble metal nanoparticles could be a powerful tool for enhancing the emission of QD glasses.

#### 3.3.2 Other Strategies to Enhance Emission of QD Glasses

In addition to the effective route detailed in the previous section, there has been some work focusing on the growth of core/shell QDs and the optimization of QD crystallization in the glass matrices, aiming to improve the quality and optical performance of QD glasses.

**Figure 8:**
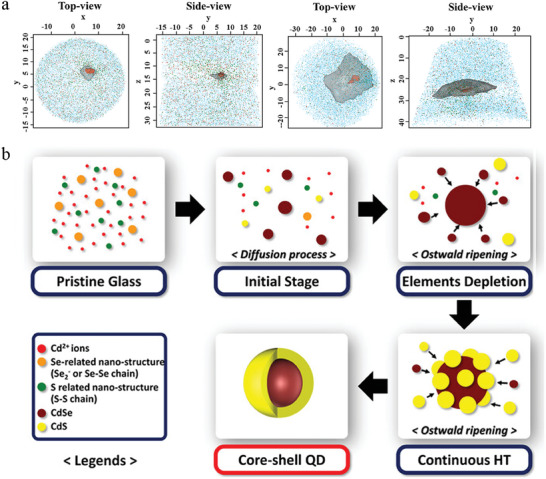
a) Iso‐surface concentration analyses of 7% Cd (red), 18 and 25% Zn (gray) from specimens irradiated for 15 min (the left top‐ and side‐view) and 60 min (the right top‐ and side‐view). The top view: xy plane; side view: yz‐plane. Reproduced with permission.^[^ ^61^ ^]^ Copyright 2023, The American Ceramic Society, John Wiley and Sons. b) Schematic illustration of the formation of CdSe/CdS core/shell QDs in silicate glass. Reproduced with permission.^[^ ^9h^ ^]^ Copyright 2020, American Ceramic Society (ACERS), John Wiley and Sons.

Growing core/shell QDs in glasses could be an effective avenue to enhance PLQY due to the combined effects of surface defect passivation and exciton confinement in the core. In 2020, Jong Heo and his coworkers prepared Cd‐Zn‐Se core/shell QDs in silicate glass using a continuous laser irradiation technique.^[^ ^61^ ^]^ As shown in **Figure** **8**a, Zn ions were incorporated into a precipitated Cd‐rich CdSe core under laser irradiation. Using a local electrode atom probe (LEAP), they visualized the 3D elemental distribution of the grown QDs in the glass, confirming the formation of QDs with a Cd‐rich core and Zn‐rich shell. Recently, more works have claimed that the core/shell QDs have been grown in glass matrices, such as CdSe/CdS QDs in silicate glass (Figure 8b),^[^ ^9^, ^62^ ^]^ CdS/ZnS QDs in borosilicate glass,^[^ ^63^ ^]^ CdSe/ Cd_1‐x_ Zn_x_Se QDs in silicate glass,^[^ ^11^, ^64^ ^]^ CdS/ Cd_1‐x_ Zn_x_S QDs in silicate glass^[^ ^65^ ^]^ and Cu^2+^ doped ZnSe/ZnS QDs in silicate glass.^[^ ^66^ ^]^ The effort to coat a shell onto the first precipitated core has gone some way to elevating the PLQY of QD glass, but there is still some way to go to match their colloidal counterparts. Although less work has been done to date on the enhancement of QD emission in NP glass matrices, as compared with dense glasses, the growing body of research of the latter is of great significance and shows promise for the potential of further optimization of QD emission in NP glass.

Promoting the diffusion of QD precursor ions in the glass matrices was found to be beneficial for improving luminescence of QD glasses. For example, introducing fluorine in the glass,^[^ ^43c^ ^]^ optimizing glass compositions^[^ ^67^ ^]^ and incorporating a nucleating agent (like Dy^3+^) in the glass matrix,^[^ ^68^ ^]^ all promote the nucleation and growth of the QDs and give rise to very high quantum yields of the QD glasses (67.14% for CdS QD glass,^[^ ^67^ ^]^ 80/60/50% for CsPbX_3_ (X = Br, (Br/I) and I) QD glasses,^[^ ^43c^ ^]^ and 31.8% for CsPbI_3_ QD glass^[^ ^68^ ^]^).

Modifications to the defect states of the grown QDs can be useful to enhance luminescence in QD glasses. TiO_2_ was incorporated along with ZnO QDs in dense glass by means of melt‐quenching and subsequent thermal treatment technique.^[^ ^69^ ^]^ It has been found that the introduction greatly impacts the concentration of non‐bridging oxygen and related defects in the glasses. The obtained ZnO QD glass exhibited green emission and yellow emission, which originated from the oxygen vacancy defects and oxygen interstitial defects, respectively. As the concentration of TiO_2_ in the system increased, the green and yellow emission first escalated and then showed a reducing tendency. Based on the above findings, the optimal concentration of TiO_2_ was found to be 1.0 mol% in the glass matrix for high fluorescence. A factor of 10 enhancement of the emission has been observed in comparison with that of TiO_2_‐free glasses. In addition, a temperature below T_g_ of the glass was chosen for the thermal treatment procedure, which ensured the small size of the grown ZnO QDs close to their exciton Bohr radius. The combined effects of the introduction of TiO2 and the controlled thermal treatment procedure resulted in the greatly enhanced emission of the ZnO QD glasses.^[^ ^69^ ^]^

Energy transfer processes from doping species in grown QD glass, as exemplified by the doping of Gd^3+^ rare earth ions to CsPbBr_3_ QD glasses, helps to enhance their optical performance.^[^ ^70^ ^]^ Doping of Gd^3+^ ions promotes the nucleation of CsPbBr_3_ QDs in the glass matrix and the Gd^3+^ ions transfer their absorbed energy to the grown QDs effectively. In addition, doping of Gd^3+^ ions contributes noticeably to the electronic density of states near the conduction band and valence band edges of CsPbBr_3_ QDs, which is believed to be helpful for emission improvement. Thanks to these combined effects, a maximum quantum efficiency of 37.7% has been achieved for CsPbBr_3_ QD glass when the concentration of the Gd^3+^ ions was fixed at 4 mol%.^[^ ^70^ ^]^

With the ever‐growing effort to enhance the optical performance of QD glasses, different types of QD glasses with high enough emission and stability for real world applications can be expected in the coming years.

## 4 Emerging Applications of QD Glasses

### 4.1 Solid State Lighting and Display

QD glasses feature outstanding mechanical strength, thermal stability and resistance to moisture compared to its colloidal counterparts, offering greater suitability for LED applications, especially white LEDs (WLED).

Not only have CdSe, CdS and Cd‐S‐Se QD glasses already been used as the color converter for LEDs,^[^ ^71^ ^]^ but CdSe/CdS core/shell QDs and other QDs have also been grown in glasses via a facile melt quenching method and employed as a complete inorganic color converter for LEDs and backlit liquid crystal displays (LCD).^[^ ^62^, ^68^, ^72^ ^]^ The color rendering index (CRI)), color correlated temperature (CCT) and chromaticity of the built WLED can be adjusted by tuning the thermal treatment duration (namely the size of the grown QDs). Compared to colloidal QDs, this complete inorganic QD color converter demonstrated higher stability without showing any practical degradation in the conversion efficiency.

**Figure 9:**
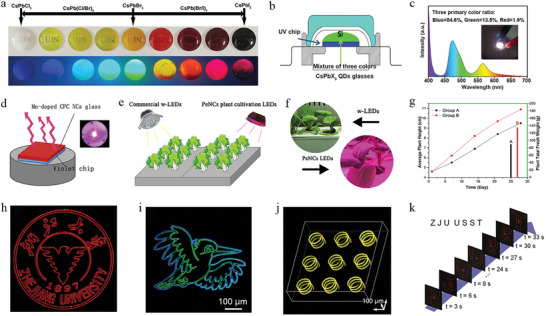
a) Photographs of CsPbX_3_ QD glasses under UV light, b) schematic of the structure of the WLED device, and c) electroluminescent (EL) spectra and photograph (inset) of the WLED device. Reproduced with permission.^[^ ^73^ ^]^ Copyright 2022, American Chemical Society. d) Schematic illustrations of the assembled LED device with the Mn‐doped perovskite QD glass and violet chip, e) the cultivation of cabbages under commercial WLEDs and the Mn‐doped perovskite QD glass based LEDs, f) photographs of the cabbages under two different irradiations and g) comparison between the average heights of the plants under different irradiations. Reproduced with permission.^[^ ^40g^ ^]^ Copyright 2020, American Ceramic Society (ACERS), John Wiley and sons. h,i) Zhejiang university logo and multicolor pattern produced with perovskite QD glass, j) 3D microhelix CsPb(Br_1‐x_I_x_)_3_ QD arrays, and k) demonstration of a dynamic holographic display (the letters on the top left are the images shown at various times), respectively. Reproduced with permission.^[^ ^11f^ ^]^ Copyright 2022, The American Association for the Advancement of Science.

Perovskite QDs, especially the inorganic perovskite QDs CsPbX_3_ (X = Cl, Br, I) feature poor moisture resistance due to their ionic nature. CsPbX_3_ QD glass showed superior moisture resistance thanks to the effective protection afforded by the zinc borosilicate glass matrix, maintaining 90% of the original PL intensity after immersion in a water for a month, which makes CsPbX_3_ QD glass quite promising for application in LEDs as shown in **Figure** **9**a–c.^[^ ^73^ ^]^ In addition, a rapid‐response and reusable LED sensor for halomethanes has been prepared by growing inorganic perovskite QDs in a NP Al_2_O_3_‐SiO_2_ glass matrix.^[^ ^14^ ^]^ The porous nature was retained after growth of the perovskite QDs, facilitating rapid permeation of the halomethanes into the NP channels. In this LED sensor, upon excitation of the blue LED chip with 395 nm emission, the CsPbBr_3_ QDs showed green emission (500 nm), transitioning immediately (within 2 s) to red photoluminescence (625 nm) after immersion in CH_3_I solution. Since the Br‐ can replace I‐ in the QDs, the halomethane LED sensor could be recovered by immersing the used sensor in a CsBr solution.

Perovskite QD glass has also been exploited for indoor plant lighting.^[^ ^40g^ ^]^ Perovskite QD glasses exhibiting 600–700 nm emission were obtained by judicious doping with Mn^2+^ ions resulting in effective energy transfer from perovskite QDs to Mn^2+^ ions, opening up a spectral window of orange‐red emission of interest to photocatalysis in plants. In this work, LEDs were assembled for the irradiation of cabbages. Figure 9d–f shows the experimental setup, in which Mn‐doped perovskite QD glass on a violet chip was used in the cultivation of cabbages alongside cabbages under the irradiation of commercial WLEDs. After 28 days of cultivation under the same ambient temperature, humidity and nutrition conditions, the average height of the plants irradiated under the home‐built Mn‐doped perovskite QD glass‐based LEDs was found to be 1.4 cm higher than that of the commercial WLED‐ irradiated cabbages (Figure 9g).^[^ ^40g^ ^]^

For perovskite QD glass prepared via ultrafast laser irradiation, not only can single color or multicolor 2D patterns be produced (Figure 9h,i), but 3D microhelix arrays can be achieved (Figure 9j). In addition, dynamic holographic display of letters “Z”, “H”, and “U” were reconstructed as shown in Figure 9k, indicating that a 3D holographic display can be realized by exciting specific perovskite QD glasses.^[^ ^11f^ ^]^

Furthermore, erasing and recovery of solid displays based on QD glasses has already been achieved. CsPbBr_3_ QDs can be grown and erased in dense glass through femtosecond laser irradiation and thermal annealing based on their ionic nature and the low required formation energy of perovskite QDs.^[^ ^73^ ^]^ The CsPbBr_3_ QDs were formed in the glass under femtosecond laser irradiation, which was followed by the absence of emission under continued laser irradiation. The repeated erasing and recovery procedures did not make a noticeable difference to the optical properties of the CsPbBr_3_ QD glass in terms of PL intensity, peak position and full width at half maximum (FWHM). Since the QDs were grown in dense glasses, both the QD glasses and the erased ones demonstrated incredible stability in both an organic and inorganic chemical environment (in ethanol and water), representing a route to integrating them in optoelectronic devices for even more promising performance.^[^ ^11c^ ^]^

### 4.2 Photocatalysis

Photocatalysis is a promising route to generate green energy and to degrade dye pollutants in rivers. QDs demonstrate suitable bandgap energy for redox reactions of hydrogen/oxygen generation, large absorption coefficient and high carrier mobility, rendering them promising candidates for photocatalysis applications. QDs including CdS, ZnS and so forth have been explored in the field of photocatalysis. However, one issue to overcome is photo‐anodic corrosion of the photocatalysts, which limits their operational lifespan for practical applications.^[^ ^6^, ^74^ ^]^ Embedding QDs in a glass offers a strategy to tackle the photo corrosion issue effectively.

**Figure 10:**
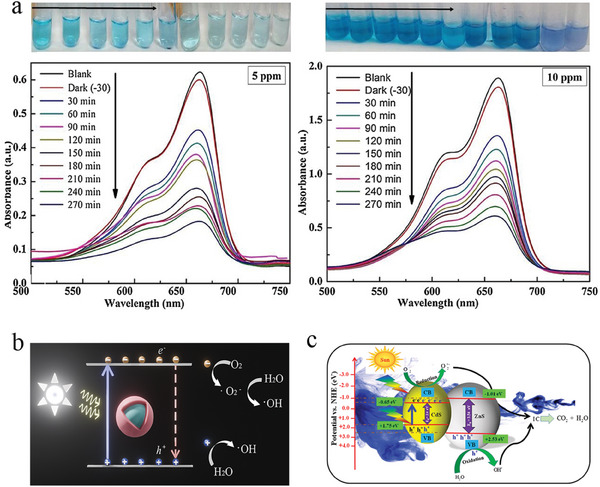
a) Photographs of the degraded methylene blue solution and the corresponding UV–vis absorption spectra (left: 5 ppm, right: 10 ppm) with CdS in borosilicate glass as the catalyst. Reproduced with permission.^[^ ^75^ ^]^ Copyright 2018, Elsevier. b,c) Schematic diagrams for the photocatalysis mechanisms of core QDs and core/shell QDs in glass matrix. Reproduced with permission.^[^ ^63^ ^]^ Copyright 2021, Elsevier.

CdS QDs have been grown in borosilicate glass via the traditional melt‐quenching and subsequent heat treatment method as photocatalyst for photodegradation of methylene blue dye.^[^ ^75^ ^]^ In this work (**Figure** **10**a), 0.1 g of CdS QD glass powder was suspended in 100 mL of aqueous solutions of methylene blue dye with different concentrations and the photocatalysis reaction started upon the exposure of the reaction system to sunlight. Photodegradation efficiency of 70.6% and 68.0% was observed for 5 and 10 ppm methylene blue aqueous solutions.^[^ ^75^ ^]^ Methyl orange can also be degraded using CdS QD glass, showing 29.4% photodegradation efficiency under 6 h of sunlight irradiation (Figure 10b).^[^ ^39c^ ^]^ Enhanced photodegradation efficiency has also been reported for indigo carmine using borosilicate glass loaded with heterostructured CdS/ZnS QDs as the photocatalyst; the photodegradation was as high as 87.2% after 6 h of sunlight irradiation (Figure 10c).^[^ ^63^ ^]^ This enhanced photodegradation efficiency can be attributed to suppression of photoexcited exciton recombination due to the possibility of interfacial charge transfer of holes from the valance band of ZnS to the valance band of CdS.

QD glass can also be employed for solar hydrogen production by decomposing hydrogen sulfide. CdS QDs were grown in sodium borosilicate glass via a melt‐quenching and subsequent heat treatment route for photo‐decomposition of H_2_S under visible light.^[^ ^6c^ ^]^ The obtained QD glass displayed improved photostability thanks to the protection of the glass matrix and the hydrogen production rate reached 3570 µmol h^−1^. This system features reusability via simple distilled water washing, producing more or less the same amount of hydrogen after three cycles of photodecomposition reaction. In another work, the CdS_0.5_Se_0.5_ and CdSe QDs were grown in germanate glass using a melt‐quenching and subsequent heat treatment method to decompose hydrogen sulfide to produce H_2_.^[^ ^76^ ^]^ The bandgap of the QDs was tuned by changing the heat treatment temperature for more efficient visible light absorption. This QD glass showed enhanced stability compared to a naked powder catalyst. Hydrogen evolution rates as high as 8164.53 and 7257.36 µmol h^−1^ g^−1^ were obtained using CdS_0.5_Se_0.5_ and CdSe QDs in germanate glass, respectively.

### 4.3 Nonlinear Optics and Ultrafast Lasers

**Figure 11:**
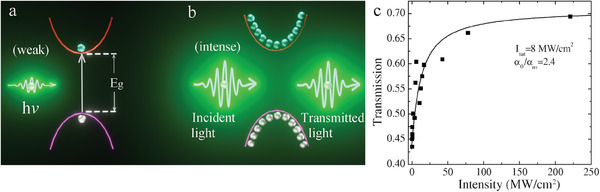
Saturable absorption induced by Pauli‐blocking. a) light with relatively weak intensity interacts with the NLO material and most light is absorbed, while b) high transmission is observed when the incident light is of such an intensity that Pauli‐blocking becomes significant. c) The transmission increases with incident laser intensity in PbS glass at a saturated intensity of 8 MW cm^−2^. Reproduced with permission.^[^ ^81^ ^]^ Copyright 2004, Elsevier.

Nonlinear optics is an important branch of optics, studying light‐matter interactions in which the response to the applied electric field is not linearly proportional to the electric field strength. It can be explained by the phenomenon of polarizability. When intense light interacts with a NLO material, negative charges (such as electrons) and positive charges (such as holes) will be separated by the optical electric force and move in opposite directions. This leads to a polarizability that can be described as: ***P *** (***r***, *t*) = ɛ_0_ ***χ*** ^(1)^ · ***E*** + ɛ_0_ ***χ*** ^(2)^: ***EE*** + ɛ_0_ ***χ*** ^(3)^⋮ ***EEE*** + ·* * · * * · * * · * * · * * ·, where ɛ_0_ represents the permittivity of free space, ***E*** (***r***, *t*), the optical induced electric field and χ^n^ is the n‐th order optical susceptibility. The NLO susceptibility χ^2^ is a second‐order NLO effect such as second‐harmonic generation (SHG), and the third‐order one is utilized to characterize third‐harmonic generation such as two‐photon absorption (TPA) and saturable absorption. These NLO phenomena have been widely reported in QDs. Sizeable SHG has been reported in core/shell CdTe/CdS QDs with a diameter of 10–15 nm that were embedded in polymer.^[^ ^77^ ^]^ TPA was observed in colloidal CdSe QDs and the TPA coefficient was found to have a linear dependence on the diameter of QDs from 2.2 to 6.8 nm.^[^ ^78^ ^]^ Saturable absorption has been demonstrated in many colloidal QDs in solution, such as CdTe/CdS core/shell colloidal QDs at a central wavelength of 515 nm^[^ ^79^ ^]^ and black phosphorus QDs in n‐methyl pyrrolidone (NMP) under irradiation by ultraviolet pulse lasers.^[^ ^80^ ^]^ Saturable absorption is an important NLO phenomenon that is explained by Pauli‐Blocking. When relatively low intensity light interacts with a NLO semiconductor, photons transfer energy to electrons in the semiconductor valence band. The electrons are excited to the conduction band, resulting in a low transmission, see **Figure** **11**a. Pauli‐Blocking refers to a situation in which a transition state is occupied and cannot accept more incoming electrons with the same quantum states. This leads to an increase in transmission through the semiconductor under intense light irradiation, see Figure 11b. Briefly, a saturable absorber absorbs weak light and allows intense light to pass through. Hence, the transmission increases as the incident intensity increases in the saturable absorption process. A typical example of this effect is shown in Figure 11c for the case of PbS QD glass.^[^ ^81^ ^]^

Saturable absorption can be described by the total absorption coefficient: α(I) = α_0_/(1+*I*/*I*_s_), where α_0_ is the linear absorption coefficient, *I* is the incident intensity and *I_s_* is saturation intensity.^[^ ^82^ ^]^ This can be employed to fit the transmission as a function of incident intensity for a saturable absorber, such as in the case of PbS QD glass as shown in Figure 11c.^[^ ^81^ ^]^ We make a summary of saturable absorption in a range of QDs embedded in glasses, see **Table** **3**. PbS QDs have been grown with different concentrations in glass, showing saturable absorption in a wide spectral range. A.A. Lagatsky et al. grew PbS QDs in silicate glass and measured its transmission as a function of laser intensity at a wavelength of 1.34 µm.^[^ ^81^ ^]^ At a central wavelength of 2.1 µm, PbS glass possesses promising nonlinear saturable absorption with a saturated intensity of 2 ± 0.2 MW cm^−2^ and a ratio of ground‐state to excited‐state absorption cross sections of 0.08 ± 0.01.^[^ ^83^ ^]^ This NLO behaver was reported in PbS QD glasses at other wavelengths such as 1.05,^[^ ^84^ ^]^ 1.064, 1.34, 1.54,^[^ ^85^ ^]^ 2.3–2.5 µm.^[^ ^86^ ^]^ It can be seen that PbS QD glasses possess saturable absorption mainly in the near‐ to mid‐ infrared region. Another case that has attracted a lot of research interest regarding the NLO response is PbSe QD glass, which has been grown for ultrafast lasers in a wide wavelength range. PbSe QDs grown in phosphate glass have been proven to have stable saturable absorption at wavelengths of 1.067, 1.351,^[^ ^85^ ^]^ and 2.1 µm.^[^ ^87^ ^]^ PbSe QDs in phosphate glass were also reported to have saturable absorption at wavelengths of 1.54 µm with a saturated intensity of ≈30 MW cm^−2^.^[^ ^85^ ^]^ In addition, many other QDs such as CdSeTe,^[^ ^88^ ^]^ CuInSSe,^[^ ^24b^ ^]^ Cu_x_Se,^[^ ^89^ ^]^ and CuGaS_2_^[^ ^15g^ ^]^ have been grown in glasses via the methods mentioned in Section 2, and have shown saturable absorption.

**Table 3:** Saturable absorption of QD glasses.

QDs	Matrix	Wavelength	α_0_	*I_S_*	Refs.
PbS	Silicate glass	1.064 µm	0.87 cm^−1^	2.3 MW cm^−2^	[90]
PbS	Glass	1.3 µm	1.34 cm^−1^	0.18 MW cm^−2^	[91]
PbS	Silicate glass	1.34 µm		8 MW cm^−2^	[81]
PbS	Silicate glass	1.34 µm	–	12 MW cm^−2^	[85]
PbS	Silicate glass	1.54 µm	≈2.5 cm^−1^	2.3 MW cm^−2^	[85]
PbS	Silicate glass	1.54 µm	≈2.5 cm^−1^	2.3 MW cm^−2^	[85]
PbS	Phosphate glass	2.09 µm	≈5 cm^−1^	2 ± 0.2 MW cm^−2^	[83, 92]
PbSe	Phosphate glass	1.54 µm	–	30 MW cm^−2^	[85]
PbSe	Phosphate glass	1.54 µm	–	30 MW cm^−2^	[85]
PbSe	Phosphate glass	2.1 µm	–	≈12 MW cm^−2^	[87b]
Cu_x_Se	Silica glass	1.08 µm	–	0.1 MW cm^−2^	[89]
CuGaS_2_	Sodium borosilicate glass	0.8 µm	–	–	[15g]

These developments bode well for NLO devices incorporating QDs, as the aforementioned high laser damage threshold of QD glasses composites is favorable to stable performance in NLO regimes which necessarily involve intense light irradiation. Research into NLO effects in nanostructures has generally focused on polymeric macromatrices such as polyvinyl acetate (PVA)^[^ ^82^ ^]^ and ionic macrocrystals (NaCl, NaBr, KCl and so forth),^[^ ^2^ ^]^ which cannot survive under long‐time intense light irradiation. In comparison to these matrices, QDs embedded in glasses possess higher thermal stability and are thus more likely to be employed in NLO devices requiring more stable operation.

To generate short and intense pulses of light, several ultrafast techniques have been developed, including Q‐switching and mode‐locking. In the Q‐switching operation, a quality factor (Q) of the cavity is tuned to change the energy that is stored in the laser cavity. The energy is built up and stored in the NLO medium of the laser when the Q factor is lowered temporarily, and is released in an intense and short laser pulse when the Q factor is enlarged quickly. In the mode‐locking operation, short laser pulses are obtained via constructive interference of different longitudinal modes that are locked in phase.

Furthermore, both Q‐switching and mode‐locking can be broken into active and passive types. In the passive type, a saturable absorber is the key photonic component in the laser cavity.

As discussed above, many QDs possess promising saturable absorption, in which most of the weak light is absorbed by the NLO material while the majority of intense light passes through it. Transmission of light in the mode‐locked laser operation can be selected by saturable absorbers.^[^ ^93^ ^]^ Hence, by incorporating QD glass as saturable absorber into the cavity of a mode‐locked laser, relatively weak light including the sideband of the pulse and continuous wave components can be absorbed, and the peak of the pulse can be kept. This results in short laser pulses.

Saturable absorbers also play a key role in passive Q‐switching lasers.^[^ ^93^, ^94^ ^]^ During the passive Q‐switching laser operation, the saturable absorber first blocks the laser light, building up energy before releasing the stored energy when it becomes saturated and allowing most of the light to pass through. A laser pulse is then generated from the released energy via modulation of the saturable absorber.

QD glasses have been incorporated into both passive Q‐switching and mode‐locked lasers, as summarized in **Table** **4**.

**Table 4:** Lasers mode‐locked or Q‐switched by QD glasses

QDs	Matrix	LMT	λ	Pulse duration	Repetition rate	Power	Active medium	Refs.
PbS	Silicate glass	Q‐switching	1.06 µm	15 ns	22.6 kHz	5.3 mW	Nd^3+^:KGd(WO_4_)_2_	[90]
PbS	Silicate glass	Q‐switching	1.9 µm	–	2.5 kHz	110 mW	Tm:KYW	[86, 95]
PbS	Silicate glass	Mode‐locking	1 µm	2.6 ps	99 MHz	250 mW	Yb:KYW	[96]
PbS	Silicate glass	Mode‐locking	1.06 µm	70 ns	90 kHz	6.0 mW	Nd^3+^:Y_3_A_l5_O_12_	[90]
PbS	Oxide glass	Mode‐locking	1207–1307 nm	4.6 ps	110 MHz	74 mW	Cr:forsterite	[91]
PbS	Phosphate glass	Mode‐locking	1.3 µm	120 ns	160 kHz	3 mW	Nd:KGW	[97]
PbS	Silicate glass	Mode‐locking	1460–1550 nm	10 ps	235 MHz	35 mW	Cr^4+^:YAG	[81]
PbS	Silicate glass	Mode‐locking	≈2 µm	980 fs	22.75 MHz	–	Tm‐Ho‐doped fiber	[92]
PbS	Phosphate glass	Mode‐locking	2.09 µm	290 ps	0.5 Hz	0.5 mJ	Cr^3+^,Tm^3+^,Ho^3+^:Y_3_Sc_2_Al_3_O_12_	[83]
CuInS_2_xSe_2(1‐x)_	Silicate glass	Mode‐locking	1.08/1.064 µm	16/36 ps	10 Hz	20/30 uJ	Nd:YAlO_3_/Nd:YAG	[24b]
Cu_x_Se	Silica glass	Q‐switching	1.067 µm	130/153 ns	≈0.4 MHz	630/780 mW	Nd^3+^:KGd(WO_4_)_2_	[89]
Cu_2_Se	Silica glass	Q‐switching	1.06/1.34/1.54 µm	100/90/60 ns	–	5/1/0.3 mJ	–	[16b]
PbSe	Phosphate glass	Q‐switching	2.1 µm	85 ns	≈0.58 Hz	22 mJ	Ho:YAG	[87b]

LMT: Laser modulation techniques.

The first fiber laser based on QD glasses as saturable absorber was reported in 2012 by R. Gumenyuk.^[^ ^92^ ^]^ In the mode‐locking operation of this fiber laser, a saturable absorber based on lead sulfide (PbS) QD glass was employed in a linear cavity with a 3 m long Tm‐Ho fiber as active medium.^[^ ^92^ ^]^ The mode‐locked laser was pumped by a 156 nm laser via 1560/1980 fiber couple combiner. PbS glass was used as both saturable absorber and the cavity mirror. A train of laser pulses at a central wavelength of ≈2 µm, with a duration of 980 fs and repetition of 22.75 MHz was obtained. It should be pointed out that soliton dynamics were studied in this fiber laser mode‐locked by the PbS QD glass, and it was found that soliton bunches form because of the slow recovery of the saturable absorber, resulting in pulse oscillations and interactions within the bunch.

In addition to this fiber laser, other reports on ultrafast lasers mode‐locked or Q‐switched by QD glasses are mainly solid‐state lasers with linear or Z‐shaped cavities. Passive mode‐locking of a neodymium (Nd) laser was achieved by using CuInSSe QD glass as saturable absorber.^[^ ^24b^ ^]^ The laser cavity was linear with a highly reflecting mirror and an output mirror of 40% transmission. By pumping the active medium with a xenon flash lamp, pulses with a duration of 16 ps at 1.08 µm and 36 ps pulses at 1.064 µm were generated for Nd:YAG and Nd:YAlO_3_, respectively.^[^ ^24b^ ^]^ A. M. Malyarevich et. al. reported a neodymium laser with a typical linear cavity, a highly reflecting mirror and another output couple cavity mirror (Reflection = 60%).^[^ ^90^ ^]^ The saturable absorber here was PbS QD silicate glass and the laser was modulated by both Q‐switching and mode‐locking techniques. Laser pulses at 1.06 µm with a duration of 15 and 70 ns, repetition rate of 22.6 kHz, output power of 5.3 and 6.0 mW were obtained from Q‐switching and mode‐locking operations, respectively.^[^ ^90^ ^]^ Another solid‐state laser with a linear cavity was modulated by Cu_x_Se QD silica glass, obtaining laser pulses with a duration of 153 nm and a peak power of 0.78 W.^[^ ^89^ ^]^ By pumping with a 802 nm diode laser, a laser Q‐switched by PbS QD obtained pulses with a pulse energy of 44 J and e repetition rage of 2.5 kHz at a central wavelength of 1.9 µm.^[^ ^86^ ^]^ Solid‐state lasers with Z‐shape cavities were also successfully modulated by PbS QD glasses.^[^ ^81^, ^91^ ^]^ The performance of these lasers modulated by QD glasses are summarized in Table 4. It can be seen that QD glasses have been employed in a list of lasers including fiber lasers, solid‐state lasers and so on. The wavelength of the output laser pulses covers a wide spectral range from about the near‐infrared to mid‐infrared.

However, it should be pointed out that there remains much scope for further research regarding QD glasses as photonic devices in ultrafast lasers. It can readily be noticed from **Table** **4** that with the exception of several Cu‐based and one case of PbSe QDs as dopant in glasses for the generation of ultrafast laser pulses, most research has focused on PbS QDs. This to some extent limits the mode‐locked lasers’ performance as regards the wavelength, pulse duration, repetition rate etc. Most ultrafast lasers based on QD glasses to date have been mainly linear cavities. Other Pulsed laser configurations have developed in recent years, such as circular cavity fiber lasers, which may benefit from saturable absorbers based on QD glass. As discussed in the previous section, many techniques can be employed to grow other QDs with outstanding NLO properties in glasses, which has huge potential for mode‐lockers.

### 4.4 Other Possible Applications of QD Glasses

In addition to the above promising applications, QD glasses have also been explored for other potential uses. For example, CdS QDs grown in dense glass using the melt‐quenching technique have been applied for the inhibition of bacterial colonies. Pure CdS QDs can be easily photo‐corroded in aqueous media containing oxygen upon exposure to visible light. Embedding QDs in glass favors their stability, especially in suppressing their photo‐corrosion rate.^[^ ^75^ ^]^ CdS QD glass prepared by melt‐quenching technique has been investigated for inhibition of both Gram‐positive and Gram‐negative bacteria. It has been found that the minimum inhibitory concentration values for both types of bacteria to inhibit 50% of isolates are 88.07 and 61.36 µg mL^−1^, respectively. This shows the great potential of CdS QD glasses as a good replacement for existing antimicrobial materials. Other potential applications like solar concentrators,^[^ ^98^ ^]^ photodetectors and X‐Ray detectors,^[^ ^99^ ^]^ lasers,^[^ ^100^ ^]^ optical filters,^[^ ^101^ ^]^ security and information storage and so forth have also been explored.^[^ ^10i^ ^]^

## 5 Conclusion and Future Outlook

In this review, we have summarized the current state‐of‐the‐art techniques to grow QDs, including, but not limited to Pb‐based, Cd‐based, and perovskite QD glasses. Both the conventional dense glass and the recently explored NP glass have been employed as matrix in which to embed QDs. Using these techniques, not only have core QDs been grown in glasses, but attempts have also been made to grow alloyed and core/shell‐structured QDs in situ, aiming to enhance the optical performance of the resulting QD glasses. Their emission range can be tuned to a certain extent by modifying certain parameters in the particular preparation technique used to grow them. The obtained QD glasses feature higher thermal and photo stability, rendering them more suitable for photocatalysis, light emitting, and photovoltaic applications.

Although extensive progress has been achieved so far in this fast‐evolving area, some challenges remain. First, the category of QDs that has been successfully grown in glasses to date are mainly heavy metal‐containing perovskite QDs (CsPbX_3_), Cd‐based and Pb‐based chalcogenide QDs (CdS, PbS, PbSe, and so forth). Even though several reports have been devoted to the preparation of Cu‐based QDs in NP glasses,^[^ ^15^, ^102^ ^]^ more effort should be made to fabricate glasses impregnated with other QDs from an already expansive list including carbon dots, Si QDs, graphene QDs, Pb‐free perovskite QDs (CsBX_3_, B = Sn, Bi, *etc*.) and other QD categories for particular applications.

Second, the morphology of QDs grown in dense and NP glass is limited to spherical QDs. The 1D (nanorod) and 3D (tetrapod) morphology would bring some unique optical properties (like polarized emission) to the obtained QD glasses, which is of particular interest in applications such as solar concentrators. For example, 3D printing is a powerful technique that can be pursued to prepare the nanoporous glasses in a programmable way regarding the pore size and pore shape.

Thus far, PLQY of the grown QDs is quite constrained in glasses, owing to the limited passivation of their surface defects. Even though much effort has been devoted to improving the PLQY of QD glasses, such systems have yet to rival their colloidal counterparts in this regard. Growing heterostructured core/shell QDs is a promising route. A clearer understanding of the (growth technique‐dependent) fundamental nucleation processes in QD glasses would help direct efforts to enhance PLQY. At the same time, new methods can be proposed to grow heterostructured QDs which favor passivation of surface defects and hence improvement of the PLQY.

Many QDs possess interesting electronic energy structures and glasses have amazing optical performance such as high laser damage threshold and high optical transmission in a wide spectral range. Hence, QD glasses are important materials for optical and photonic applications particularly NLO devices like laser mode‐lockers. As discussed above, research on ultrafast lasers based on QD glasses has focused mainly on PbS and PbSe QDs, whose optical response is mainly in the range from near‐ to mid‐ infrared. This results in mode‐locked or Q‐switched lasers that operate at wavelengths of ≈1 to ≈2 µm. To extend the application of QDs in the field of NLO, the first thing to be done is to develop a greater compositional range of QDs embedded in both dense and NP glasses, including but not limited to Cu‐based, carbon and perovskite QDs. Facilitating the use of such materials would open up a wider spectral range to ultrafast lasers. Other methods to enhance the applicability of QD glasses as saturable absorbers are to tune the diameters of QDs and to grow alloyed or core/shell QDs in glasses. Following from this, the next step would be to fabricate QD glasses with excellent NLO properties into general photonic devices. For instance, a highly reflective thin film can be coated onto the surface of QD glasses that possess saturable absorption, forming saturable absorption mirrors (SAMs) based on QD glasses.

Notwithstanding the challenges outlined above, rapid development of this research area indicates the exciting potential of solid‐state QD glasses. The many examples included in this review attest to the progress that has been made in harnessing the unique properties of pristine QDs and turning them toward real‐world applications. As this gap continues to be bridged, the promise of enhanced stability and superior optical properties offered by QDs embedded in dense and NP glasses represents an exciting opportunity.

## Conflict of Interest

The authors declare no conflict of interest.

## Author Contributions

X.B. drafted the original manuscript. G.W., Y.K.G., J.J.M., and B.J. checked and edited the final manuscript.
